# Time to Treatment With Nivolumab or Pembrolizumab for Patients With Advanced Melanoma in Everyday Practice

**DOI:** 10.7759/cureus.19835

**Published:** 2021-11-23

**Authors:** Doran Ksienski, Pauline T Truong, Nicole S Croteau, Angela Chan, Eric Sonke, Tiffany Patterson, Melissa Clarkson, Mary Lesperance

**Affiliations:** 1 Medical Oncology, British Columbia Cancer Agency, Victoria, CAN; 2 Radiation Oncology, British Columbia Cancer Agency, Victoria, CAN; 3 Anesthesiology, Pharmacology & Therapeutics, University of British Columbia, Victoria, CAN; 4 Medical Oncology, British Columbia Cancer Agency, Surrey, CAN; 5 Internal Medicine, University of British Columbia, Victoria, CAN; 6 Clinical Trials Unit, British Columbia Cancer Agency, Victoria, CAN; 7 Mathematics and Statistics, University of Victoria, Victoria, CAN

**Keywords:** time to treatment, melanoma, nivolumab, pembrolizumab, immunotherapy

## Abstract

Background

The anti-programmed cell death one antibodies (Anti-PD-1 Ab) pembrolizumab or nivolumab are commonly prescribed to patients with advanced melanoma. The purpose of the current study is to identify baseline clinical characteristics associated with time to treatment initiation (TTI) of pembrolizumab or nivolumab for advanced melanoma and whether treatment delays are associated with differences in survival outcomes.

Methods

All patients receiving Anti-PD-1 Ab as a first-line treatment for advanced melanoma outside of clinical trials at British Columbia Cancer Agency between 10/2015 and 10/2019 were identified retrospectively. TTI was defined as the interval from pathologic diagnosis of advanced melanoma to first Anti-PD-1 Ab treatment. To determine the association between TTI and baseline characteristics, multivariable Cox proportional hazard regression analyses provided an estimate of the instantaneous relative risk of starting treatment at any time point (hazard ratio [HR] >1 indicates shorter TTI). To describe changes in overall survival (OS) observed for each four-week delay in treatment initiation, multivariable cox proportional hazard regression modelling was also performed.

Results

In a cohort of 302 patients, the median TTI was 52 days (interquartile range 30.2-99.0). Pulmonary metastases (M1b)/non-central nervous system visceral metastases (M1c) vs. metastases to skin or non-regional lymph nodes (M1a)(HR=1.50, 95% CI=1.12-2.02; p=0.007) and pre-treatment Eastern Cooperative Oncology Group Performance Status (ECOG PS) >1 (vs 0/1, HR=1.50, 95% CI= 1.11-2.01; p=0.008) were associated with earlier TTI. An association between treatment delay and improved OS was observed.

Conclusion

Patients having visceral metastases and poor baseline ECOG PS were more likely to initiate Anti-PD-1 Ab sooner. The association of shorter TTI with worse OS likely represents confounding by indication (urgent treatment offered to patients with aggressive disease).

## Introduction

In 2020, an estimated 1300 Canadians died from advanced melanoma [1]. Melanoma typically arises from melanocytes in cutaneous locations, but uveal, acral, mucosal, and unknown primary melanoma are the recognized subtypes [2]. Historically, metastatic melanoma had a guarded prognosis with a 25% one-year overall survival (OS) rate [3]. The prospect of long-term survival is now possible for a subset of patients due to the availability of immune checkpoint inhibitors and targeted therapy for tumors harboring a BRAF V600 mutation.

First-line immunotherapy treatment options for patients with advanced melanoma include monotherapy with anti-programmed cell death-1 (Anti-PD-1) antibodies such as nivolumab or pembrolizumab and combination treatment with nivolumab and ipilimumab (a cytotoxic T lymphocyte antigen-4 (CTLA-4) antibody.) Median OS in KEYNOTE-006 (a phase III study of pembrolizumab versus ipilimumab) and CheckMate 066 (a phase III study of nivolumab versus dacarbazine chemotherapy) was approximately three years. Importantly, four to seven percent of trial participants developed severe immune-related adverse events (irAE) [4-5]. In the phase III CheckMate 067 study, four cycles of nivolumab and ipilimumab followed by maintenance nivolumab resulted in a median OS of six years [6]; high grade irAE were documented in 42% of patients [7].

Given mounting external pressures on healthcare systems, understanding factors influencing the timeliness of cancer care and the impact of treatment delays on outcomes are critical. It is possible that patient populations underrepresented (i.e., individuals aged 75 years and older) or excluded (i.e., patients with poor Eastern Cooperative Oncology Group performance status, ECOG PS) from clinical trials might experience longer waiting times to start immunotherapy. Hanna et al. demonstrated poorer survival outcomes with each four-week delay in the initiation of adjuvant systemic therapy for breast, colon, and rectal cancer [8]. To our knowledge, no population-based studies have explored the relationship between time to treatment initiation (TTI) of Anti-PD-1 antibodies for advanced melanoma and OS.

In this multicentre retrospective analysis, we examine patient- and tumor- level characteristics associated with TTI of Anti-PD-1 antibodies. In addition, we describe differences in survival outcomes associated with delays in initiation of immunotherapy.

## Materials and methods

Data source and patient population

We performed a multicenter retrospective analysis of patients receiving pembrolizumab or nivolumab monotherapy as a first-line treatment for advanced melanoma (unresectable stage III or IV as defined by the American Joint Committee on Cancer, Cancer Staging Manual, Eighth Edition) [9] at the six regional centers of British Columbia (BC) Cancer Agency between 10/2015 and 10/2019. Data cut-off was 2/2021. Patients receiving Anti-PD-1 antibodies as part of a clinical trial or in combination with ipilimumab were excluded. Patients were identified using the BC Cancer Agency pharmacy database. A search of the BC Cancer Agency pharmacy database identified a total of 449 patients with advanced melanoma who received nivolumab or pembrolizumab during the studied time interval. 147 patients were excluded as nivolumab was given concurrently with ipilimumab (95 patients) or Anti-PD-1 antibody was administered in the second-line setting or greater (52 patients). As such, 302 patients met the inclusion criteria.

Outcome measures

The primary endpoint was the association of baseline patient- and tumor-level covariates with TTI (defined as the time from pathologic diagnosis of advanced melanoma to first Anti-PD-1 antibody treatment). The associations of OS and time to treatment failure (TTF) for each four-week treatment delay were secondary outcomes. OS was defined as the time from initiation of Anti-PD-1 antibody therapy until death from any cause or censored at last follow-up. TTF was defined as the time interval from first Anti-PD-1 antibody treatment to initiation of another line of systemic therapy or death (whichever came first), with censoring at the time of the last follow-up.

Clinical variables

Patient-level pre-treatment variables extracted by chart review included: age at Anti-PD-1 antibody initiation (categorized as less than or at least 75 years old), gender, population size of primary residence (small or medium population size: less than 100,000 people; large population size: at least 100,000 people) [10], ECOG PS, Charlson Comorbidity Index (CCI, not including the presence of metastatic melanoma) [11], serum lactate dehydrogenase (LDH) with an upper reference limit of 244U/L, absolute neutrophil count (N), and absolute lymphocyte count (L). elevated serum LDH and serum N/L>4.9 are negative prognostic factors of patients with advanced melanoma receiving Anti-PD-1 antibody monotherapy [12]. Tumor-specific variables included melanoma subtype, stage, and presence of BRAF V600 mutation. 

Statistical analysis

Descriptive statistics were calculated for clinicopathologic characteristics. Median follow-up time was calculated using the reverse Kaplan-Meier method [13]. Univariable (UVA) and multivariable (MVA) Cox proportional hazard (PH) regression models were used to generate point estimates of the hazard ratio (HR) and the corresponding 95% confidence interval (CI) for pre-treatment variables and TTI. In these models, the event of interest is treatment receipt, and thus an HR>1 implies a higher “hazard” of treatment and, therefore, shorter TTI.

Time-dependent MVA Cox PH models provided point estimates of the HR and 95% CI for each four-week increase in treatment delay. The ‘waiting time paradox’ is a type of confounding by indication due to the inclusion of patients with very aggressive disease who present early yet still have a short survival [14-15]. In an attempt to minimize bias related to the waiting time paradox and also for patients with a very indolent disease, UVA and MVA Cox PH models of OS were constructed that excluded patients who died within eight weeks of diagnosis of advanced melanoma and those who started Anti-PD-1 antibody monotherapy more than one year after diagnosis. 

All p-values were based on two-sided hypothesis tests, and those less than 0.05 were considered statistically significant. Statistical analyses were performed using R version 4.1.0, packages survival version 3.2, survminer version 0.4.9, and gtsummary version 1.4.2 (The R Foundation, Vienna, Austria) [16-20]. This research was approved by the University of British Columbia Research Ethics Board.

## Results

Study population

Table 1 describes the baseline characteristics of the 302 patients meeting inclusion criteria. 41.7% of the cohort were aged at least 75 years, 20.5% were ECOG PS 2 or greater, 42.0% had an elevated LDH, and 25.1% had blood N/L >4.9

**Table 1:** Baseline characteristics and treatment received Abbreviations: CCI - Charlson Comorbidity Index; ECOG PS - Eastern Cooperative Oncology Group performance status; IQR - interquartile range; LDH - lactate dehydrogenase; M1a - metastases to skin or distant lymph nodes; M1b - pulmonary metastases; M1c - non-central nervous system (CNS) visceral metastases; M1d - CNS metastases; n - number of patients, Anti-PD-1 Ab - anti-programmed cell death 1 antibody

	Whole cohort n = 302
Characteristic	n (%)
Age in years, Median (IQR)	72 (64 – 80)
Gender	
Male	186 (62%)
Female	116 (38%)
Population size of primary residence	
<100,000 people	161 (53%)
≥100,000 people	141 (47%)
ECOG PS	
0	90 (29.8%)
1	150 (49.7%)
2	50 (16.6%)
3	11 (3.6%)
4	1 (0.3%)
CCI, Median (IQR)	1 (0 – 2)
Melanoma subtype	
Cutaneous	232 (77%)
Mucosal	22 (7.3%)
Ocular	22 (7.3%)
Unknown Primary	26 (8.6%)
Stage	
III/M1a	68 (23%)
M1b	49 (16%)
M1c	152 (50%)
M1d	33 (11%)
BRAF V600 mutation	77 (25%)
LDH >224	127 (42%)
Neutrophil/Lymphocytes ≥5	76 (25%)
Anti-PD-1 Ab	
Nivolumab	42 (14%)
Pembrolizumab	260 (86%)

Regarding tumor characteristics, most patients had cutaneous melanoma (76.8%), were BRAF V600 wild type (74.5%), and had non-central nervous system (CNS) visceral metastases (M1c, 50.3%). Of the 33 patients who presented with metastases to the CNS (M1d) prior to initiation of immunotherapy, 17 patients underwent whole-brain radiotherapy (WBRT), five patients received stereotactic radiotherapy (SRT), one patient underwent neurosurgery followed by SRT, and one patient underwent neurosurgery followed by WBRT. The remaining 27.3% (9/33) of patients with M1d stage started immunotherapy with untreated brain metastases.

TTI Analysis

The Median TTI of Anti-PD-1 antibodies for the whole cohort was 52.0 days (interquartile range, 30.2-99.0.) On multivariable analysis, pre-treatment pulmonary (M1b) or non-CNS visceral metastases (M1c) (p=0.007) and Eastern Cooperative Oncology Group Performance Status (ECOG PS) >1 (p=0.008) were associated with earlier TTI (Table 2)

**Table 2:** Cox proportional hazard regression analysis of factors associated with time to treatment initiation In this model, the hazard ratio provides the instantaneous relative risk of starting anti-programmed cell death 1 antibodies at any given time point (n=302). Abbreviations: CCI - Charlson Comorbidity Index; CI - confidence interval; ECOG PS - Eastern Cooperative Oncology Group performance status; HR - hazard ratio; IQR - interquartile range; LDH - lactate dehydrogenase; M1a - metastases to skin or distant lymph nodes; M1b - pulmonary metastases; M1c - non-central nervous system (CNS) visceral metastases; M1d - CNS metastases; n - number of patients; REF - reference

	Univariable			Multivariable		
Variable	HR	95% CI	p-value	HR	95% CI	p-value
Age						
<75	REF	REF		REF	REF	
≥75	0.84	0.66 – 1.06	0.14	0.87	0.68 – 1.11	0.3
Gender						
Male	REF	REF		REF	REF	
Female	0.79	0.63 – 1.00	0.053	0.81	0.63 – 1.03	0.080
Population size of primary residence						
<100,000 people	REF	REF		REF	REF	
≥100,000 people	0.86	0.69 – 1.08	0.2	0.91	0.72 – 1.15	0.4
ECOG PS						
0/1	REF	REF		REF	REF	
≥2	1.55	1.16 – 2.05	0.003	1.50	1.11 – 2.01	0.008
CCI (continuous)	1.05	1.00 – 1.10	0.072	1.04	0.98 – 1.09	0.2
Melanoma subtype						
Cutaneous	REF	REF		REF	REF	
Non-cutaneous	1.06	0.81 – 1.39	0.7	1.02	0.77 – 1.34	>0.9
Stage						
III/M1a	REF	REF		REF	REF	
M1b/M1c	1.45	1.09 – 1.92	0.011	1.50	1.12 – 2.02	0.007
M1d	1.46	0.96 – 2.23	0.080	1.35	0.85 – 2.12	0.2
BRAF V600 mutation	1.31	1.00 – 1.70	0.047	1.31	0.98 – 1.75	0.064
LDH > 224	1.21	0.96 – 1.53	0.10	1.04	0.82 – 1.32	0.7
Neutrophil/Lymphocytes ≥ 5	1.22	0.94 – 1.58	0.14	1.07	0.81 – 1.41	0.7

Survival Analysis

The median follow-up for the whole cohort was 29.9 months. At the last follow-up, 137 patients (45.4%) were still alive. Median OS for the whole cohort was 22.5 months (95% CI, 18.3-29.5 months.) Factors associated with shorter OS on multivariable analysis for the whole cohort were pre-treatment ECOG PS >1 (p<0.001), CNS metastases (M1d) (p=0.003), serum LDH >244 (p<0.001), and a serum N/L >4.9 (p=0.002) (Table 3).

**Table 3:** Cox proportional hazard regression analysis of factors associated with overall survival (n =302) Abbreviations: CCI - Charlson Comorbidity Index; CI - confidence interval; ECOG PS - Eastern Cooperative Oncology Group performance status; HR - hazard ratio; IQR - interquartile range; LDH - lactate dehydrogenase; M1a - metastases to skin, lymph nodes; M1b - pulmonary metastases; M1c - non-central nervous system (CNS) visceral metastases; M1d - CNS metastases; REF - reference

	Univariable			Multivariable		
Variable	HR	95% CI	p-value	HR	95% CI	p-value
Age						
<75	REF	REF		REF	REF	
≥75	1.15	0.84 – 1.57	0.4	1.33	0.96 – 1.86	0.088
Gender						
Male	REF	REF		REF	REF	
Female	0.93	0.68 – 1.27	0.6	1.06	0.77 – 1.47	0.7
Population size of primary residence						
<100,000 people	REF	REF		REF	REF	
≥100,000 people	0.92	0.68 – 1.25	0.6	1.01	0.74 – 1.39	>0.9
ECOG PS						
0/1	REF	REF		REF	REF	
≥2	2.40	1.70 – 3.38	<0.001	2.23	1.56 – 3.19	<0.001
CCI (continuous)	1.04	0.97 – 1.11	0.3	1.01	0.94 – 1.08	0.9
Melanoma subtype						
Cutaneous	REF	REF		REF	REF	
Non-cutaneous	1.11	0.78 – 1.59	0.5	1.01	0.69 – 1.46	>0.9
Stage						
III/M1a	REF	REF		REF	REF	
M1b/M1c	1.98	1.28 – 3.08	0.002	1.53	0.97 – 2.41	0.068
M1d	3.21	1.82 – 5.65	<0.001	2.46	1.36 – 4.44	0.003
BRAF V600 mutation	1.00	0.70 – 1.42	>0.9	1.11	0.77 – 1.61	0.6
LDH > 224	2.20	1.62 – 2.99	<0.001	2.14	1.56 – 2.94	<0.001
Neutrophil/Lymphocytes ≥ 5	2.25	1.63 – 3.13	<0.001	1.77	1.24 – 2.52	0.002

Each four-week delay in treatment initiation was associated with improved OS (HR=0.96, 95% CI=0.95-0.97, p<0.001) (Table 4).

**Table 4:** Time-dependent Cox proportional hazard regression analysis of factors associated with overall survival including time to treatment initiation (TTI) of anti-programmed cell death 1 antibodies (n =302) Abbreviations: CCI - Charlson Comorbidity Index; CI - confidence interval; ECOG PS - Eastern Cooperative Oncology Group performance status; HR - hazard ratio; IQR - interquartile range; LDH - Lactate dehydrogenase; M1a - metastases to skin or distant lymph nodes; M1b - pulmonary metastases; M1c - non-central nervous system (CNS) visceral metastases; M1d - CNS metastases; n - member of patients; REF - reference

	Univariable			Multivariable		
Variable	HR	95% CI	p-value	HR	95% CI	p-value
Age						
<75	REF	REF		REF	REF	
≥75	1.15	0.92 – 1.44	0.2	1.24	0.98 – 1.58	0.074
Gender						
Male	REF	REF		REF	REF	
Female	0.88	0.70 – 1.10	0.3	0.99	0.79 – 1.24	>0.9
Population size of primary residence						
<100,000 people	REF	REF		REF	REF	
≥100,000 people	0.91	0.73 – 1.13	0.4	1.01	0.81 – 1.27	>0.9
ECOG PS						
0	REF	REF		REF	REF	
≥1	1.77	1.37 – 2.28	<0.001	1.54	1.17 – 2.02	0.002
CCI	1.05	1.00 – 1.10	0.059	1.01	0.96 – 1.06	0.8
Melanoma subtype						
Cutaneous	REF	REF		REF	REF	
Non-cutaneous	1.15	0.90 – 1.48	0.3	1.13	0.86 – 1.47	0.4
Stage						
III/M1a	REF	REF		REF	REF	
M1b/M1c	2.04	1.49 – 2.78	<0.001	1.65	1.20 – 2.27	0.002
BRAF V600 mutation	1.04	0.81 – 1.33	0.8	1.23	0.95 – 1.61	0.12
LDH > 224	2.25	1.81 – 2.79	<0.001	2.03	1.62 – 2.53	<0.001
Neutrophil/Lymphocytes ≥ 5	2.22	1.76 – 2.79	<0.001	1.78	1.39 – 2.28	<0.001
TTI, per month (time dependent)	0.96	0.95 – 0.97	<0.001	0.96	0.95 – 0.97	<0.001

MVA, including 280 patients (10 patients who died within eight weeks of diagnosis and 12 patients with TTI >1 year were excluded), confirmed a positive association between treatment delay per four-week increment and OS (HR=0.96, 95% CI=0.95-0.97, p<0.001).

Median TTF for the whole cohort was 13.9 months (95% CI, 9.4-20.2). On multivariable analysis including all 302 patients, no association was observed between each four-week treatment delay and TTF (HR=0.98, 95% CI 0.96-1.01, p=0.3).

## Discussion

The main objectives of this retrospective study were to identify baseline factors associated with TTI of first-line Anti-PD-1 antibody monotherapy for advanced melanoma and to describe a potential association between treatment delay and survival outcomes. We found that patients with visceral metastases (M1b/M1c stage) and ECOG PS of at least two were more likely to have a shorter TTI. Furthermore, a four-week treatment delay was associated with a lower risk of death.

Survival outcomes for patients with advanced melanoma have greatly improved over the past decade due to the use of immune checkpoint inhibitors in routine practice. A meta-analysis of 42 phase II trials of metastatic melanoma conducted between 1975 to 2005 determined a median OS in enrolled patients of six months [3]. In contrast, the median OS for patients in registration trials of Anti-PD-1 antibody monotherapy was approximately three years [4-5]. Receipt of combination nivolumab and ipilimumab in CheckMate 067 yielded a median OS of six years. Importantly combination immunotherapy carries a significant risk of severe irAE compared to single-agent Anti-PD-1 antibody treatment (42% and four to seven percent, respectively) [4-5,7]. As such, pembrolizumab or nivolumab monotherapy is typically prescribed to patients unable to tolerate or unwilling to accept the higher toxicity of combination therapy.

Despite these revolutionary advances in systemic therapy, significant knowledge gaps exist regarding optimal TTI of immunotherapy. In the context of early-stage melanoma, Conic et al. noted a higher risk of mortality amongst patients with stage I melanoma who received definitive surgery >30 days after an initial biopsy [21]. To our knowledge, there are no Canadian or international guidelines providing benchmarks for TTI from the date of diagnosis of advanced melanoma. In the current series median TTI of Anti-PD-1 antibody monotherapy was 52 days. Using the National Cancer Database, Dobry et al. determined that the median TTI of any type of immunotherapy for patients diagnosed with advanced melanoma in the United States was also approximately 52 days [22].

Early initiation of immunotherapy for advanced melanoma should intuitively be associated with better outcomes. Longer times to treatment could be detrimental in several ways: greater disease-related morbidity, deterioration of ECOG PS, and patient anxiety [15]. In the current analysis, treatment delays (measured in four-week intervals) were observed to be associated with longer OS. This likely relates to the waiting time paradox, a form of confounding by indication: patients with aggressive disease are treated within a shorter time yet ultimately still have a poor prognosis [14,15]. For instance, we found that patients with visceral disease (M1b/M1c stage) were likely to initiate treatment sooner than those with metastatic disease limited to the skin and non-regional lymph nodes (M1a). Liver metastases are common in advanced melanoma and are associated with poor response to immunotherapy [23]. Specifically, in a retrospective analysis of 357 patients with metastatic cutaneous melanoma receiving single or double agent immunotherapy, those with liver metastases and elevated serum LDH had a median OS of 4.2 months [24]. As such, it is possible that patients presenting with symptomatic liver metastases began immunotherapy treatment soon after diagnosis yet still experienced poor survival outcomes.

Advanced melanoma has a predilection for central nervous system (CNS) metastases, with approximately 1/3 of patients presenting with brain metastases. Consistent with prior studies, the M1d stage in the current series was associated with a poor prognosis [22]. Local therapies such as surgery or radiation have traditionally been prioritized due to limited CNS penetration of chemotherapeutic agents. Delay in the initiation of immunotherapy amongst patients with M1d disease is likely also attributable to the need for steroid taper for symptomatic CNS disease or as part of radiotherapy protocols. In the current series, the majority of patients with the M1d stage underwent local therapies prior to starting immunotherapy. A number of recent phase II trials, typically involving good ECOG PS individuals with asymptomatic brain metastasis, have demonstrated intracranial responses with Anti-PD-1 antibody monotherapy [25], combination Anti-PD-1 and CTLA-4 antibodies [26], and combination BRAF/MEK inhibitors [27]. Further research will be required to identify patient subgroups able to initiate systemic therapy prior to local modalities in order to treat both intra- and extracranial disease as soon as possible.

In the current series, older age at diagnosis of advanced melanoma (defined as an age of at least 75 years) was not associated with delayed TTI of Anti-PD-1 antibody monotherapy. This is encouraging as age-based differences in the management of melanoma are thought to contribute to poorer outcomes in older individuals. For instance, older patients are less likely to undergo wide local excision or a sentinel lymph node biopsy [28]. In the metastatic setting, advanced age has been associated with a decreased likelihood of receiving immunotherapy [22]. Clinicians might be more willing to prescribe Anti-PD-1 antibodies to patients aged 75 years or greater (despite being underrepresented in clinical trials) due to a growing number of retrospective studies demonstrating comparable toxicity in older and younger patients [29].

It is noteworthy that pretreatment ECOG PS of at least two was associated with shorter TTI. Poor ECOG PS, as confirmed in our multivariable analysis, is an established negative prognostic factor. Since KEYNOTE-006 and CheckMate 066 only included patients with ECOG PS 0/1, whether ECOG PS >1 predicts for lack of benefit from immunotherapy is unclear. According to American Society of Clinical Oncology guidelines, if poor ECOG PS is a result of tumor burden (as opposed to comorbidities), then there is a potential therapeutic role for immune checkpoint inhibition [30]. Importantly as supportive care medications such as steroids are often withheld inpatient receiving immunotherapy, prospective studies in this patient population are urgently required.

The present analysis has several limitations, including patient and treatment selection biases inherent in retrospective studies. We focused on the interval from diagnosis to first treatment; however, the diagnostic interval (i.e., time from the first symptom to diagnosis) might also affect survival outcomes. Second, it is likely that the association between TTI and OS is complex, and the current analysis was underpowered to completely describe this relationship. Last, our findings likely only pertain to individuals receiving frontline Anti-PD-1 antibody monotherapy and not to combination immunotherapy.

## Conclusions

In this study of patients receiving Anti-PD-1 antibody monotherapy as an initial treatment for advanced melanoma, TTI was shorter for those with visceral disease and poor ECOG PS. A four-week delay in treatment initiation was associated with improved survival, which likely reflects triaging of symptomatic patients to be started on treatment sooner. Given the growing number of patients with advanced melanoma receiving immunotherapy, further study into the association between treatment delays and survival outcomes is needed.
